# Correction: Dendritic fibrous nanosilica activated bovine excrement fiber to enhance the initial characteristics and durability of concrete

**DOI:** 10.1039/d5ra90082d

**Published:** 2025-06-23

**Authors:** Mohsen Karimi, Seyed Mojtaba Movahedifar, Amin Honarbakhsh, Mahdi Nobahari, Rahele Zhiani

**Affiliations:** a Department of Civil Engineering, Neyshabur Branch, Islamic Azad University Neyshabur Iran movahedi_far@yahoo.ca amin_honarbakhsh@yahoo.com; b New Materials Technology and Processing Research Center, Department of Civil Engineering, Neyshabur Branch, Islamic Azad University Neyshabur Iran; c Department of Chemistry, Neyshabur Branch, Islamic Azad University Neyshabur Iran; d New Materials Technology and Processing Research Center, Department of Chemistry, Neyshabur Branch, Islamic Azad University Neyshabur Iran; e Advanced Research Center for Chemistry, Biochemistry and Nanomaterial, Neyshabur Branch, Islamic Azad University Neyshabur Iran

## Abstract

Correction for ‘Dendritic fibrous nanosilica activated bovine excrement fiber to enhance the initial characteristics and durability of concrete’ by Mohsen Karimi *et al.*, *RSC Adv.*, 2025, **15**, 3979–3987, https://doi.org/10.1039/D4RA05716C.

The authors regret that the authors' affiliations were incorrectly shown in the original manuscript. The corrected list of affiliations is as shown herein.

The Royal Society of Chemistry apologises for these errors and any consequent inconvenience to authors and readers.

